# 5-Aminolevulinic Acid-Squalene Nanoassemblies for Tumor Photodetection and Therapy: In Vitro Studies

**DOI:** 10.1186/s11671-017-2408-y

**Published:** 2018-01-11

**Authors:** Andrej Babič, V. Herceg, E. Bastien, H.-P. Lassalle, L. Bezdetnaya, Norbert Lange

**Affiliations:** 1 School of Pharmaceutical Sciences, University of Geneva, Rue Michel Servet 1, 1211 Geneva 4, Switzerland; 2 School of Pharmaceutical Sciences, University of Lausanne, Lausanne, Switzerland; 3 Centre de Recherche en Automatique de Nancy (CRAN), CNRS UMR 7039 (Centre National de la Recherche Scientifique), Université de Lorraine, Campus Sciences, Vandœuvre-lès-Nancy, France; 4 Research Department, Institut de Cancérologie de Lorraine, Avenue de Bourgogne, 54519 Vandœuvre-lès-Nancy, France

**Keywords:** 5-Aminolevulinic acid, Nanoassemblies, Fluorescence, Photodetection, Photodynamic therapy

## Abstract

Protoporphyrin IX (PpIX) as natural photosensitizer derived from administration of 5-aminolevulinic acid (5-ALA) has found clinical use for photodiagnosis and photodynamic therapy of several cancers. However, broader use of 5-ALA in oncology is hampered by its charge and polarity that result in its reduced capacity for passing biological barriers and reaching the tumor tissue. Advanced drug delivery platforms are needed to improve the biodistribution of 5-ALA. Here, we report a new approach for the delivery of 5-ALA. Squalenoylation strategy was used to covalently conjugate 5-ALA to squalene, a natural precursor of cholesterol. 5-ALA-SQ nanoassemblies were formed by self-assembly in water. The nanoassemblies were monodisperse with average size of 70 nm, polydispersity index of 0.12, and ζ-potential of + 36 mV. They showed good stability over several weeks. The drug loading of 5-ALA was very high at 26%. In human prostate cancer cells PC3 and human glioblastoma cells U87MG, PpIX production was monitored in vitro upon the incubation with nanoassemblies. They were more efficient in generating PpIX-induced fluorescence in cancer cells compared to 5-ALA-Hex at 1.0 to 3.3 mM at short and long incubation times. Compared to 5-ALA, they showed superior fluorescence performance at 4 h which was diminished at 24 h. 5-ALA-SQ presents a novel nano-delivery platform with great potential for the systemic administration of 5-ALA.

## Background

Medical nanotechnology has introduced promising new drug delivery platforms. They are composed from biocompatible and biodegradable nanomaterials that help to improve chemical stability and pharmacokinetic profile of pharmacologically active compounds while providing a controlled delivery at the site of action [1–3]. However, only few nanoparticle systems have so far reached the market. The main pitfalls of existing nanoparticles (NPs) are mainly their poor drug loading (usually less than 5%) and “burst release effect” which brings about a premature release of significant portion of the drug before reaching the target site. This causes adverse side effects and might lead to toxicity and loss of pharmacological activity [4].

Squalene (SQ) is a linear triterpene with the chemical formula C_30_H_50_ and a precursor of cholesterol and other steroids [5]. In the human body, squalene is synthesized in the liver and in the skin and transported by low density lipoprotein (LDL) and very low density lipoprotein (VLDL) in the blood [6]. In the context of tumor therapy, squalene exhibited a strong potentiation effect on certain chemotherapeutic agents [7]. Because it is widely found in nature and safe, squalene has found its applications in pharmaceutical technology as an excipient in the preparation of lipid emulsions for the delivery of vaccines, various active compounds, and genes [6, 8, 9]. Squalene has been found suitable for the covalent conjugation to different drugs. Advanced nanosystems created this way incorporate squalene conjugated to chemotherapeutic agents like gemcitabine [10–12], paclitaxel [13], cisplatin [14], or doxorubicin [15]. This approach is called “squalenoylation” and involves prodrug strategy with the formation of the nano-colloidal systems where the active principle is covalently bound [16, 17]. Squalene-based nanoassemblies (NAs) are formed by self-assembly of functional components in aqueous media and are characterized by inherent high drug loading [18]. Squalenoylation has been found to enhance drug stability and increase the solubility of poorly water-soluble drugs, hence improving bioavailability and prolonging drug half-life in the systemic circulation [14, 16]. In most cases, such self-assembled NAs display better pharmacological activity than the parent drug [16, 19]. In addition, squalenoylation provides a mean to construct NAs containing both a therapeutic and an imaging modality [20]. Similar theranostic NAs have been reported by Couvreur and co-workers by incorporating a MRI agent into squalenoyl-gemcitabine (SQgem) nanoassemblies [14]. These types of multifunctional systems may be of a major importance in developing new theranostic agents for personalized medicine.

**Fig. 1:**
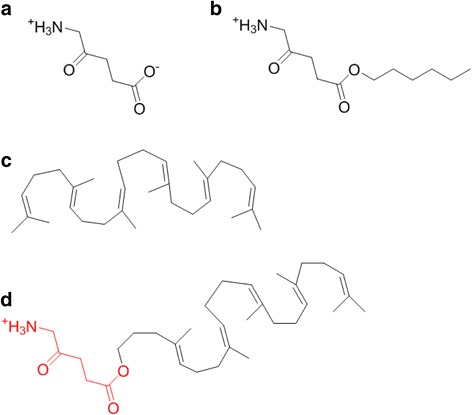
Chemical structures of NA building block elements. 5-ALA (**a**), 5-ALA-Hex (**b**), squalene (**c**), and 5-ALA-SQ (**d**)

In the context of cancer theranostics, the administration of a small molecule 5-aminolevulinic acid (5-ALA) (Fig. 1) has led to the clinical use of 5-ALA for photodynamic therapy (PDT) [21–23], photodiagnosis (PD) [24], and fluorescence-guided tumor resection in brain cancer glioma patients [25–27]. The theranostics is achieved by the metabolism of 5-ALA and selective accumulation of protoporphyrin IX (PpIX) within the cancer tissue as a consequence of the bypassed feedback inhibition of the heme cycle [22]. However, the efficacy of 5-ALA PD and PDT is seriously hampered by its zwitterionic nature at neutral pH found in the bloodstream. Different attempts have been made to improve 5-ALA’s stability and pharmacokinetic profile. Both the amino- and the carboxylic-end of 5-ALA have been modified by various approaches [28]. The esterification of 5-ALA’s carboxyl group has led to 5-ALA methyl ester (Metvix®) [29] and is used in the topical treatment of actinic keratosis, basal cell carcinoma, and acute acne. Hexyl ester of 5-ALA (5-ALA-Hex) (Hexvix®) has gained marketing authorization for the photodiagnosis (PD) of bladder cancer [30, 31] and is experimentally exploited for the treatment of cervical cancer and severe acne [32–34].

However, with the exception of Gliolan™, the clinical use of 5-ALA and its derivatives is mostly limited to topical administration. This limits their use in more important types of cancer, such as breast, colorectal, lung, and prostate cancer. Attempts to broaden the use of 5-ALA has amino-end modified phosphatase-sensitive derivatives of 5-ALA that have shown very promising activity recently [35, 36]. Furthermore, attempts have been undertaken to encapsulate 5-ALA into different nanosystems including polymeric NAs [37–39] or liposomes [40–43] and its conjugation into dendrimers [44, 45] or gold NPs [46, 47]. Although some of these solutions have helped to improve 5-ALA stability and its pharmacokinetic profile, none of the aforementioned attempts have resulted in a successful clinical candidate in the field of cancer nanomedicine.

The aim of this work was the design and synthesis of 5-ALA-squalene (5-ALA-SQ) conjugate building block (Fig. 1d) that self-assembles in aqueous media and contains high drug loading of 5-ALA, a prerequisite for pharmacological activity in cancers. The NAs were tested on two different cancer cell lines for their fluorescence PD capabilities. Combined with recent reports of squalene-based NAs exploiting plasma lipoproteins to achieve indirect cancer targeting [48], this 5-ALA nanotechnology approach expands the use of 5-ALA for PD and PDT of different cancers, such as prostate cancer used in this study.

## Results and Discussion

### 5-ALA-SQ Building Block Synthesis

**Fig. 2:**
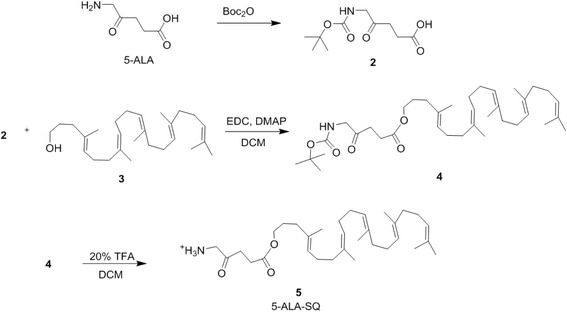
5-ALA-SQ building block synthesis. 5-ALA-SQ was synthesized in four synthetic steps using convergent synthesis from 5-ALA and SQ

An efficient converging chemical strategy was used to synthesize the 5-ALA-SQ building block from squalene and 5-ALA (Fig. 2). 5-ALA was first protected at the amino end with *N*-Boc protecting group using standard conditions. Nanoassembly-inducing squalene alcohol (**3**) was synthesized from squalene in four synthetic steps according to procedures described in the literature [49]. Boc-5-ALA (**2**) and squalene alcohol (**3**) were then coupled in the presence of EDC and DMAP to yield the protected ester derivative (**4**) in good yield. The final Boc deprotection had to be performed under mild acidic conditions to avoid electrophilic addition onto the squalene scaffold. The final product was purified by reverse-phase HPLC to yield the NA building block in good yield.

### 5-ALA-SQ Nanoassemblies

In order to achieve an efficient delivery of an active compound by nanoparticles (NPs) to its site of action, parameters such as nanoparticle size, shape, and surface charge play an important role and govern the pharmacokinetics of nano-delivery systems in the body [50]. In particular, particle size governs several pharmacokinetic phenomena such as NP half-life in the systemic circulation, sequestration by macrophages, and the extravasation through leaky vasculature into the site of action [51]. Shape and size of nanoparticles regulate their ability to extravasate through the fenestrations found in the vasculature [50, 51]. Size and shape are also very important for active targeting and uptake into cells since smaller NP and spherical shapes have a smaller surface area, thus much limited contact points in comparison to larger non-spherical nanoparticulate systems [51].

**Fig. 3:**
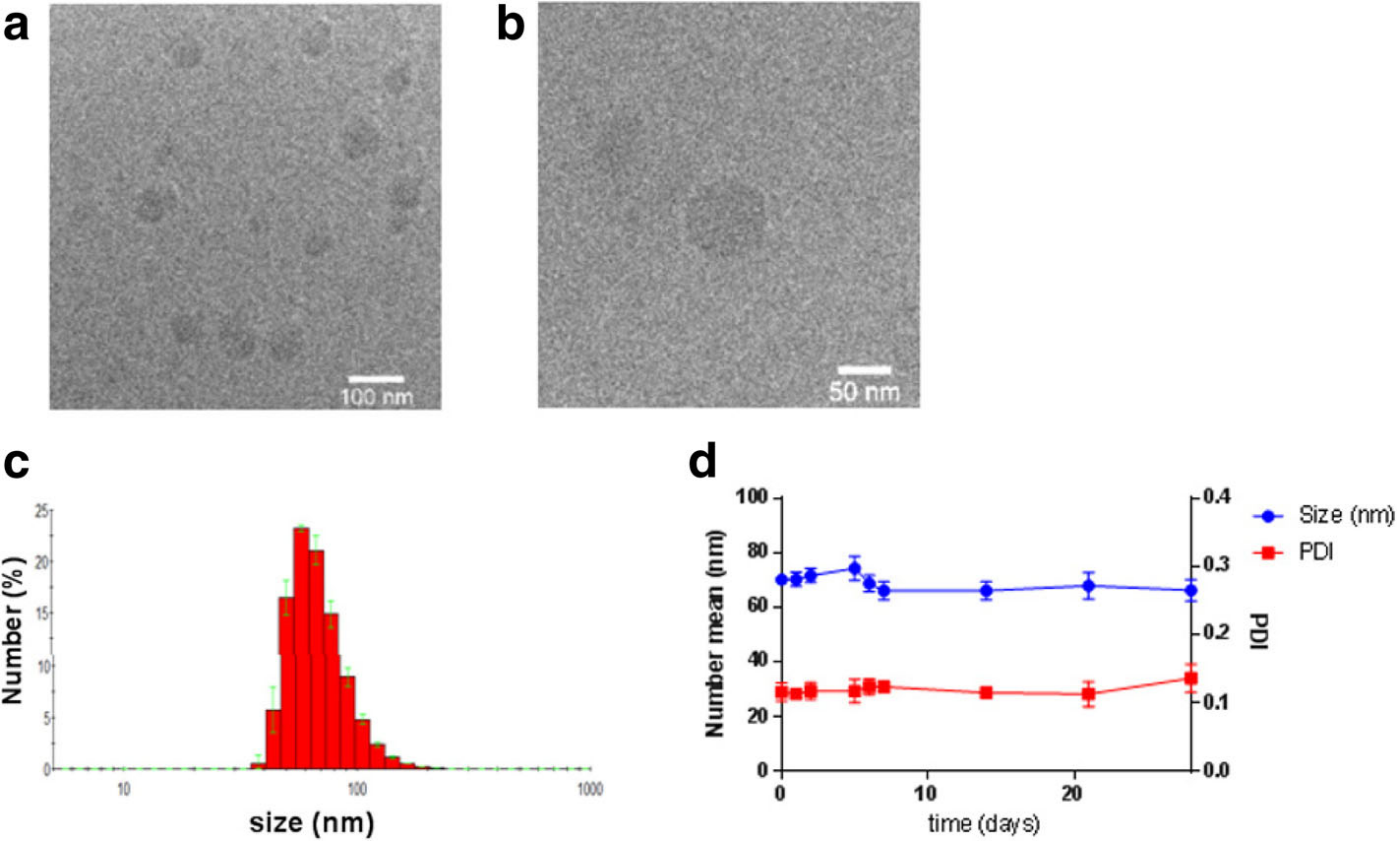
Characterization of 5-ALA-SQ NAs. Low (**a**) and high (**b**) magnification cryo-TEM images, DLS analysis (**c**), and stability at 4 °C (**d**)

The self-assembly of 5-ALA-SQ building block occurred spontaneously in aqueous media. The NAs were formed by nanoprecipitation. 5-ALA-SQ conjugate was dissolved in organic solvents and was added dropwise into water. Total evaporation of the organic solvents yielded an aqueous solution of NAs. 5-ALA-SQ NAs were monodisperse with low polydispersity index of 0.12. Electrophoretic mobility measurements gave ζ-potential of 36 mV, and dynamic light scattering (DLS) revealed monodisperse distribution of NAs with an average size of 70 nm (Fig. 3). The NA size range offers good potential for prolonged circulation in the bloodstream [50]. Nevertheless, 5-ALA-SQ NAs are significantly smaller than previously reported SQ composites which range between 100 and 300 nm indicating a different supramolecular arrangement [19].

The smaller size might be due to positively charged amino-groups and relatively small 5-ALA molecule in comparison to SQ moiety. It has been demonstrated that the variation of small molecules attached to squalene introduces changes in self-assembly and packing of compound-squalene conjugates consequently altering the shape and size of NAs [16]. It is possible that the highly positively charged amino group of 5-ALA orients itself toward the bulk water while the lipophilic chains occupy the interior of these NAs; however, the exact supramolecular structure remains to be elucidated. Despite the significantly smaller size compared to other squalene nanocomposites, the NAs display excellent shelf stability with size and PDI remaining constant over several weeks.

Another important aspect of the new 5-ALA-SQ NAs is that they achieve a drug loading of 26% which is high in comparison to other reported 5-ALA nanoparticulate systems where the loading was much less efficient [37, 38, 41]. Drug loading is very important in NP delivery because in poor drug-loaded NPs, administered dose might not be sufficient for reaching pharmacologically active concentration in target tissues [4]. The loading could be determined by simple calculation taking into account the molecular weights of 5-ALA and 5-ALA-SQ since 5-ALA is covalently bound to the squalene scaffold in 1:1 molar ratio.

### PpIX Fluorescence Kinetic Measurements in Cancer Cells

**Fig. 4:**
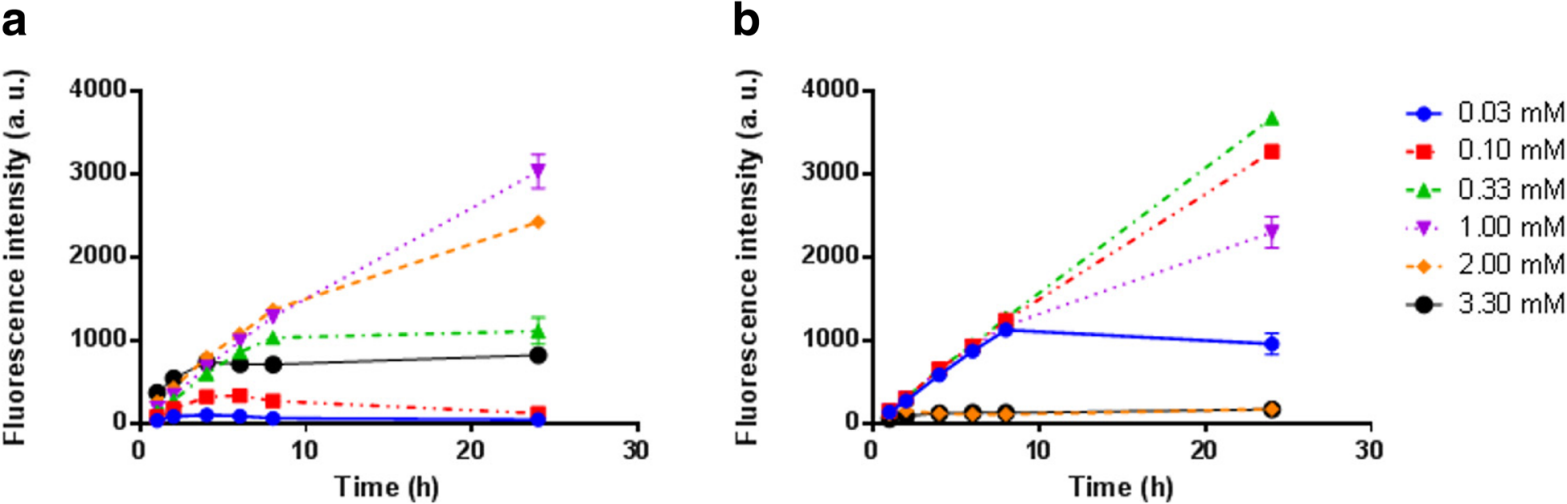
Kinetic fluorescence measurements of PpIX accumulation in PC3 cells. The cells were incubated with increasing concentrations of 5-ALA-SQ NAs (**a**) or 5-ALA-Hex (**b**)

Time-dependent formation of PpIX was evaluated in PC3 human prostate cancer cells and U87MG glioblastoma incubated with 5-ALA-SQ NAs and 5-ALA-Hex as reference. Figure 4 presents the PpIX formation in PC3 human prostate cancer cells exposed to increasing concentrations of 5-ALA-SQ NAs or 5-ALA-Hex over 24 h.

Concentration-dependent PpIX fluorescence profiles were observed for 5-ALA-SQ NAs. At 1.0 and 2.0 mM, PpIX fluorescence increased steadily over 24 h while reaching a plateau after 8 h of incubation for lower concentrations. On the other hand, 5-ALA-Hex induced the highest accumulation of PpIX in lower concentration range between 0.10 and 0.30 mM as reported previously [35, 52]. However, 5-ALA-Hex at concentrations above 1 mM was found to be toxic to cells which reduces the overall fluorescence observed and impedes its use [36].

**Fig. 5:**
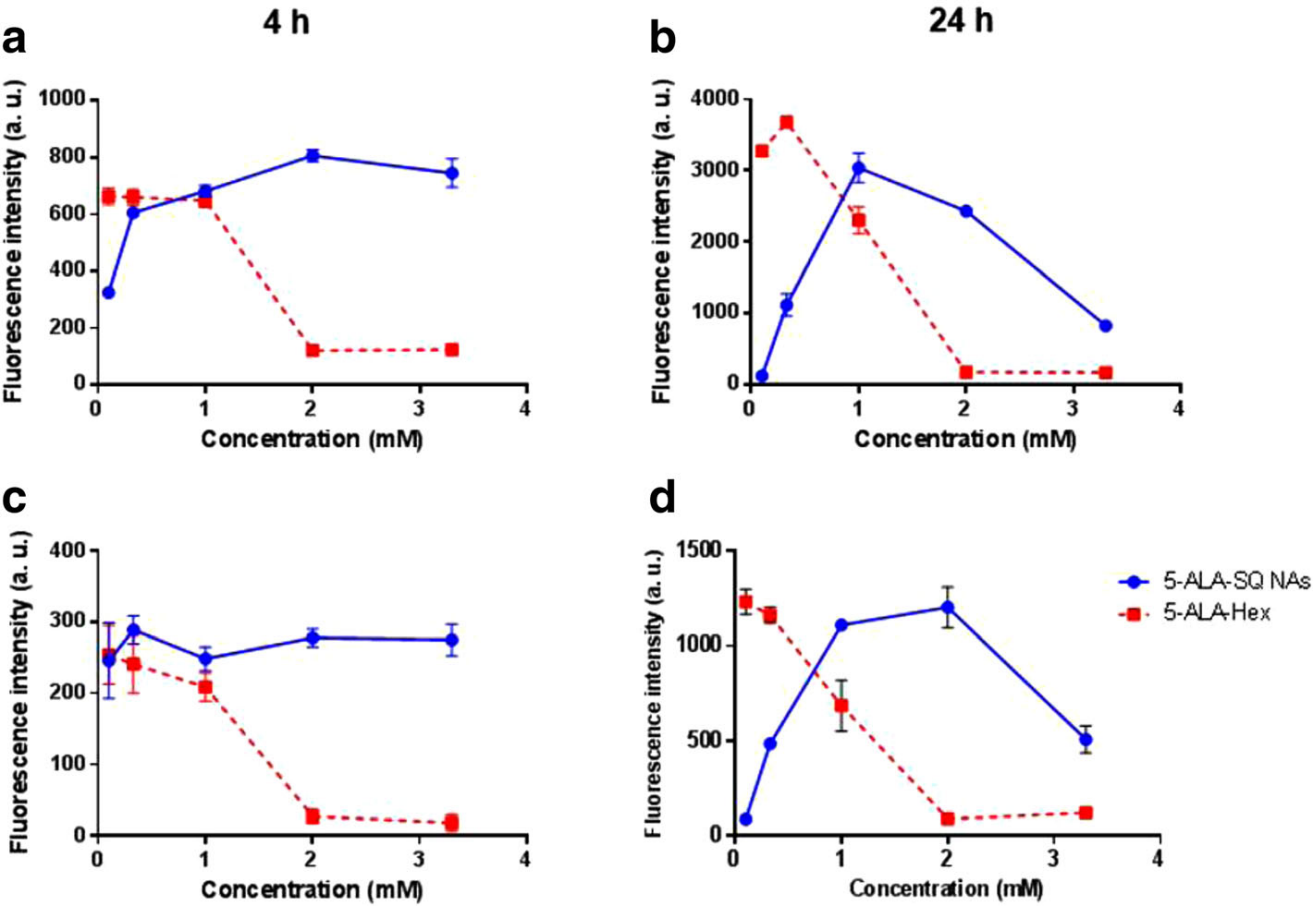
Dose-response curves. Concentration-dependent PpIX accumulation with 5-ALA-SQ NAs (blue) and 5-ALA-Hex (red) in PC3 (**a**, **b**) and U87MG (**c**, **d**) cells after 4 h (left) and 24 h (right) incubation

Next, dose-dependent PpIX accumulation in PC3 and U87MG human glioblastoma cancer cells was performed with the goal of estimating optimal NA dosing. Fluorescence intensity at 4 and 24 h of incubation with 5-ALA-SQ NAs and 5-ALA-Hex is shown in Fig. 5. Very importantly, 5-ALA-SQ NAs induced the PpIX production in both cell lines. Furthermore, maximum fluorescence levels are comparable with ALA-Hex control in both cell lines. It can also be noted that in PC3 cells, 1.0 and 2.0 mM concentrations of 5-ALA-SQ NAs were optimal for inducing the highest accumulation of PpIX. In U87MG cells, there was no significant differences between different concentrations of 5-ALA-SQ NAs for short incubation times (Fig. 5c). At 24 h, PpIX accumulation was found to be dependent on the concentration of 5-ALA-SQ NAs or 5-ALA-Hex similar to PC3 cells. A decrease in PpIX induction somewhat similar to ALA-Hex was found after longer period of incubation at higher concentrations of 5-ALA-SQ NAs which were more sensitive to the presence of NAs.

Figure 5 demonstrates that at 24 h, PpIX production curves are bell-shaped in both PC3 and U87MG cells. While 1 mM concentration of 5-ALA-SQ NAs induced the highest PpIX accumulation in PC3 cells, U87MG cells tolerated higher concentrations and the increase in PpIX fluorescence was seen up to 2 mM 5-ALA-SQ NAs. In general, higher concentrations of 5-ALA-SQ NAs were needed to efficiently induce the biosynthesis of PpIX when compared to 5-ALA-Hex, presumably due to the different ester bond cleavage rates within the cancer cells. Decrease in PpIX production was observed when concentrations higher than 1 mM of 5-ALA-Hex were used due to the non-specific toxicity of 5-ALA-Hex.This effect was much less pronounced for the 5-ALA-SQ where the fluorescence levels started to drop off only at the highest tested concentration (3.3 mM) and prolonged incubation times (Fig. 5b, d). However, the fluorescence levels were similar for both compounds without any fluorescence lag observed for the NAs.

**Fig. 6:**
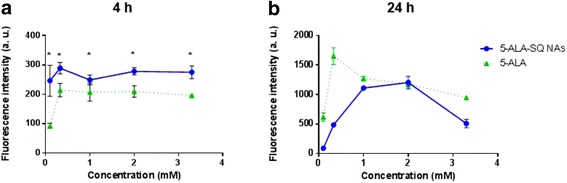
Dose-response curves. Concentration-dependent PpIX accumulation in U87MG cells after 4 h (**a**) and 24 h (**b**) incubation with 5-ALA-SQ NAs (blue) and 5-ALA (green)

The NAs were also assayed in U87MG glioblastoma against 5-ALA control which is marketed for PD of glioblastoma during surgical resection. Figure 6 presents the fluorescence of U87MG cell after 4 and 24 h. 5-ALA-SQ NAs induced significantly higher fluorescence after 4 h compared to 5-ALA which is highly relevant in a clinical setting. At 24 h, the fluorescence profile shifts in favor of 5-ALA at lower concentrations as the slow, active uptake of 5-ALA affords sufficient 5-ALA quantities within the cells. NAs still demonstrate similar fluorescence levels to 5-ALA at optimal 1.0 and 2.0 mM concentrations of NAs (Fig. 6b).

In clinical practice, 5-ALA is administered either topically or orally, but because of its charged nature, only small portion of the initial dose enters into target cells via endogenous peptide transporters like PEPT1, PEPT2, or BETA transporters, depending on the cell type [40, 53]. Recent studies on NAs from a SQgem derivative indicate that the cell entry is governed by albumin-enhanced diffusion of single-molecule building blocks and it was found to be highly dependent on presence of extracellular proteins [54, 55]. Furthermore, far-red fluorescent NAs we reported recently also demonstrated rapid internalization, and 5-ALA-SQ fluorescence kinetic experiments and the dose-response curves corroborate single-molecule building block internalization and efficient subsequent metabolism to yield fluorescent PpIX.

## Conclusions

In this in vitro proof-of-concept study, a converging chemical strategy was used to synthesize the 5-ALA-SQ building block from squalene and 5-ALA. The 5-ALA-SQ NAs were prepared by spontaneous nanoprecipitation in water. NAs were monodisperse and stable with size average of 70 nm, polydispersity index of 0.12, positive ζ-potential of 36 mV, and high, 26% 5-ALA loading. PpIX production was evaluated in vitro in two cancer cell lines by measuring the fluorescence increase over time and compared to 5-ALA and 5-ALA-Hex. The results showed that SQ-ALA NAs are very efficient in inducing the PpIX production in PC3 and U87MG cancer cell types. They outperform 5-ALA-Hex in fluorescence induction at higher concentrations at 4 and 24 h incubation times in vitro; however, compared to 5-ALA, they show superior fluorescence induction at shorter incubation times. In scope with these findings, we can conclude that 5-ALA-SQ NAs present an attractive nanotechnology solution for overcoming the pharmacokinetic drawbacks of 5-ALA. Further, in vivo experiments will evaluate their potential for the systemic delivery of 5-ALA for fluorescence PD and PDT therapy of tumors.

## Methods/Experimental

Reagents were purchased from commercial suppliers Sigma-Aldrich (Bucks, Switzerland) and Acros Organics (Basel, Switzerland) and used without further purification. Deuterated NMR solvents were obtained from Cambridge Isotope Laboratories (Tewksbury, USA). Tetrahydrofurane (THF) and dichloromethane (CH_2_Cl_2_) were obtained from an Anhydrous Engineering alumina column-based drying system. All other solvents used were HPLC grade. N,N-dimethylformamide (DMF), methanol (CH_3_OH), diethyl ether (Et_2_O) and acetone were purchased from Sigma-Aldrich (Buchs, Switzerland). Ethyl acetate (AcOEt) was purchased from Biosolve (Dieuze, France); acetonitrile (CH_3_CN) was supplied by Carlo Erba Reagents (Balerna, Switzerland). Hexane ≥ 95% of n-hexane was purchased from Fisher Chemical (Basel, Switzerland). The water used for the preparations was deionized by a Milli-Q lab water system (Millipore, Molsheim, France). Chemical reactions were performed using standard syringe-septa with positive pressure of argon to ensure anhydrous conditions.

Thin layer chromatography (TLC) was performed with aluminium-backed silica plates (Merck-Keiselgel 60 F254) with a suitable mobile phase and was visualized using a UV fluorescence lamp (254 and 366 nm) and/or developed with ninhydrine, 20% sulfuric acid, or phosphomolybdic acid (PMA). Flash chromatography was performed on an automated PuriFlash® 4100 machine from Interchim (Montlucon, France) using Interchim silica columns puriFlash® HP 30 μm equipped with a PDA detector (200–800 nm) and automated fraction collector. The elution profile was monitored using Flash Interchim software version 5.0x. Semi-preparative HPLC column was conducted on a Waters Symmetry 300TM - 5 μm (19 × 150 mm), C8 column (Baden-Dättwil, Switzerland). Analytical UPLC was conducted using Macherey-Nagel EC50/2 Nucleodur Gravity 1.8 μm column (50 × 2.1 mm) fitted on a water system equipped with a Waters PDA detector (Baden-Dättwil, Switzerland). Buffer A = CH_3_CN + 0.1% Formic acid) and buffer B = H_2_O + 0.1% Formic acid. Flow rate = 400.0 μL/min at 25 °C. ^1^H and ^13^C NMR spectra were recorded on Varian Gemini 300 MHz, Varian Innova 500 MHz, or Bruker Avance III Cryo 600 MHz spectrometers at 298 K. Chemical shifts (δ) are quoted in parts per million (ppm) and coupling constants (J) are in hertz (Hz). s for singlet, d for doublet, dd for doublet of doublets, t for triplet, q for quartet, and m for multiplet. Residual solvent peaks were used as the internal reference for the proton and carbon chemical shifts. NMR spectra were processed with Mnova version 10.0.2 software package. Low resolution mass spectrometry (LRMS) was carried out on a HTS PAL-LC10A – API 150Ex instrument in ESI (positive mode). High-resolution mass spectrometry (HRMS) was carried out on a QSTAR Pulsar (AB/MDS Sciex) instrument in ESI (positive mode). Chemical structures were drawn and named according IUPAC nomenclature using ChemBioDraw Ultra version 14.0.0.117 software package. The pH was measured on a Metrohm 691 pH meter using a spearhead electrode (Zofingue, Switzerland), calibrated with Metrohm buffers. Statistical analyses were performed using GraphPad Prism 6, 2016, (GraphPad Software) software. *P* value < 0.05 was considered as statistically significant.

### Synthesis of SQ-ALA Building Block

#### 5-(tert-Butoxycarbonylamino)-4-oxopentanoic Acid (2)

Boc-5-ALA was synthesized according to published procedure. The spectroscopic data are identical with the literature [56]. ^1^H NMR (600 MHz, DMSO-d6) δ 12.12 (s, 1H), 7.06 (t, J = 5.9 Hz, 1H), 3.76 (d, J = 5.9 Hz, 2H), 2.61 (t, J = 6.6 Hz, 2H), 2.40 (t, J = 6.5 Hz, 2H), 1.38 (s, 9H). ^13^C NMR (151 MHz, DMSO) δ 206.62, 174.07, 156.21, 78.54, 49.97, 40.38, 40.24, 40.11, 39.97, 39.83, 39.69, 39.55, 34.22, 28.64. [M+H]^+^ 232.1, found 232.7.

#### (4E,8E,12E,16E)-4,8,13,17,21-pentamethyldocosa-4,8,12,16,20-pentaen-1-ol (3)

Squalene alcohol **3** was synthesized from squalene in four synthetic steps in 23.7% yield as colorless oil according to the reported procedures [49]. ^1^H NMR (300 MHz, CDCl_3_) δ 5.17-5.06 (m, 5H, CH), 3.62 (q, *J* = 6.3 Hz, 2H, CH_2_-OH), 2.17 – 1.92 (m, 18H, CH_2_), 1.67 (s, 3H, CH_3_), 1.59 (m, 17 H, CH_3_ and CH_2_). ^13^C NMR (75 MHz, CDCl_3_) δ 135.35, 135.17, 135.14, 134.81, 131.49, 125.05, 124.64, 124.60, 124.47, 63.07, 39.98, 39.95, 39.89, 36.24, 30.92, 28.48, 26.98, 26.88, 26.78, 25.94, 17.92, 16.28, 16.23, 16.08. LRMS (ESI): m/z calculated for [M+NH_4_]^+^ 404.4, found 404.8.

#### (4E,8E,12E,16E)-4,8,13,17,21-pentamethyldocosa-4,8,12,16,20-pentaen-1-yl 5-((tert-butoxy carbonyl)amino)-4-oxopentanoate (4)

Squalene alcohol (**3**) (100 mg, 0.26 mmol), EDC (74 mg, 0.38 mmol) and DMAP (94 mg, 0.78 mmol), and 5-(tert-Butoxycarbonylamino)-4-oxopentanoic acid (2) (77 mg, 0.34 mmol) were dissolved in DCM (15 mL). After stirring overnight at ambient temperature, the solvent was evaporated under reduced pressure and crude product purified by Flash chromatography using DCM/ethyl acetate (EA) gradient giving colorless oil (108 mg, 0.18 mmol, 70%). ^1^H NMR (300 MHz, CDCl_3_) δ 5.31 – 5.20 (br s, 1H), 5.13 – 5.07 (m, 5H), 4.09 – 3.95 (m, 4H), 2.75 – 2.53 (m, 4H), 2.02 – 1.95 (m, 20H), 1.64 (s, 3H), 1.63 – 1.50 (m, 19H), 1.41 (s, 9H). ^13^C NMR (75 MHz, CDCl3) δ 204.46, 172.65, 135.30, 135.16, 135.09, 133.75, 131.43, 125.34, 124.60, 124.57, 124.48, 124.45, 64.81, 50.53, 39.96, 39.93, 39.87, 35.92, 34.56, 28.52, 28.47, 28.43, 28.24, 28.02, 26.97, 26.86, 25.92, 23.11, 17.90, 16.25, 16.21, 16.06. LRMS (ESI): m/z calculated for [M+NH_4_]^+^ 617.5, found 617.8.

#### Trifluoroacetic acid salt of 5-amino-(((4E,8E,12E,16E)-4,8,13,17,21-pentamethyldocosa-4,8,12,16,20-pentaen-1-yl)oxy)-4-oxopentanoate (5)

Compound **4** (34 mg, 57 mmol) was dissolved in DCM (2.0 mL). Trifluoroacetic acid (TFA) (200 μL) was added and the reaction mixture stirred at ambient temperature. After 10 min, the solvents were evaporated in vacuo at low temperature and traces of TFA were removed by co-evaporation with EA (3 × 10 mL). The crude product was purified by RP-HPLC using full H_2_O/AcN (0.025% TFA) gradient yielding colorless oil (25 mg, 74%). %). ^1^H NMR (300 MHz, CDCl_3_) δ 5.31 – 5.20 (br s, 1H), 5.13 – 5.07 (m, 5H), 4.09 – 3.95 (m, 4H), 2.75 – 2.53 (m, 4H), 2.02 – 1.95 (m, 20H), 1.64 (s, 3H), 1.63 – 1.50 (m, 19H). LRMS (ESI): m/z calculated for [M+H]^+^ 500.4, found 500.6.

### Preparation of Nanoassemblies

NAs were prepared by nanoprecipitation described in detail elsewhere [20]. Briefly, building block **5** (1.2 mg, 2.0 μmol) was dissolved in a 50/50 *V*/*V* mixture acetone/ethanol (500 μL). The organic phase was then added dropwise using a micro-syringe into MilliQ water (1.25 mL) at 100 μL/min under magnetic stirring. After 5 min under stirring, the magnetic stir bar was removed and the organic solvents and the excess of water removed using a rotary evaporator at 30 °C. The final concentration of nanoassemblies was 2.00 mM.

### Characterization of 5-ALA-SQ NAs

5-ALA loading of 5-ALA.SQ NA was calculated from the respective contributions of the molecular weights of 5-ALA and of 5-ALA-SQ conjugate as follows [19]:

$$ \mathrm{Loading}\ \left(\%\right)=\frac{\mathrm{MW}\ \left(5-\mathrm{ALA}\right)}{\mathrm{MW}\left(5-\mathrm{ALA}-\mathrm{SQ}\right)}\times 100 $$

Hydrodynamic diameter of NAs was measured by dynamic light scattering (DLS) using a NANO ZS instrument from Malvern (Worcestershire, UK) running the ZetaSizer 7.01 software. The analyses were performed with 4 mW He-Ne Laser (633 nm) at scattering angle of 173° at 25 °C in polystyrene (PS) micro cuvette from Brand (Wertheim, Germany). Zeta potential (ZP) was determined using the same Nano ZS instrument from Malvern in folded capillary cells DTS 1070 from Malvern. Size distribution and size mean diameter were calculated from the data. The stability of NAs stored at 4 °C was assayed by DLS at regular time points over a period of 1 month.

The morphology of NAs was assessed by cryogenic transmission electron microscope (cryo-TEM) using TECNAI® G^2^ Sphera microscope (FEI, Thermo Fisher Scientific) equipped with 2000 by 2000 pixel high resolution digital camera TCL (Gräfelfing, Germany). The vitrified ice samples were prepared using the Virtobot cryo-plunger (FEI, Thermo Fisher Scientific). NAs (2.0 μL, 2.0 mM) were applied to Quantifoil Cu/Rh 200 mesh R3.5/1 grids (SPI, West Chester, USA) and vitrified using liquid ethane.

### Cell Culture

Human prostate cancer cells PC3 (ATTC® CRL-1435™) and human glioblastoma cells U87MG (ATTC® HTB-14™) were grown and maintained by serial passage in F-12K nutrient mix (21127-022, Thermo Fisher Scientific) or minimum Essential Media (31095-029, Thermo Fisher Scientific), respectively. Cell media were supplemented with fetal calf serum (10%, CVFSVF00-01, Eurobio), streptomycin (100 μL/mL), and penicillin (100 IU/mL, 15140-122, Thermo Fisher Scientific). Cells were cultivated at 37 °C in a humidified atmosphere containing 95% air and 5% CO_2._

### In Vitro PpIX Fluorescence Kinetic Measurements

Human prostate cancer cells PC3 (12,000 cells/well) and glioblastoma cells U87MG (10,000 cells/well) were seeded in 96-well plates (clear bottom black plate, 3603, Corning). The next day, cells were exposed to increasing concentrations of 5-ALA-SQ NAs, 5-ALA-Hex, and 5-ALA in serum-free media. PpIX fluorescence was recorded with a plate reader (Safire, Tecan, Switzerland) at different time points. Excitation wavelength was set to 405 nm and emission wavelength to 630 nm. Mean values and s.d. for each concentration at each time point per plate were subtracted with the reference value (no treatment) and plotted for each cell line.
